# Comparing Deep Learning Models for Identifying Maxillary Transverse Deficiency from Intraoral Photographs

**DOI:** 10.1016/j.identj.2026.109689

**Published:** 2026-06-19

**Authors:** Jianing Li, Rui Wang, Zhou Yi, Jianhui Ni, Zhigang Zuo, Yue Wang

**Affiliations:** a Department of Orthodontics, Tianjin Medical University School and Hospital of Stomatology & Tianjin Key Laboratory of Oral Soft and Hard Tissues Restoration and Regeneration, Tianjin, PR China; b Tianjin Medical University Institute of Stomatology, Tianjin, PR China; c Department of Electrical and Computer Engineering, Faculty of Engineering, The University of Hong Kong, Hong Kong, PR China; d Department of Stomatology, The First Affiliated Hospital, Hengyang Medical School, University of South China, Hengyang, Hunan, PR China

**Keywords:** Artificial intelligence, Deep learning, Maxillary transverse deficiency, Intraoral photographs, Cone-beam computed tomography

## Abstract

**Introduction and aims:**

Maxillary transverse deficiency (MTD) is conventionally evaluated using cone-beam computed tomography (CBCT), which entails increased radiation exposure, cost, and clinical workload. Frontal intraoral photographs are routinely obtained in orthodontic practice. Using CBCT-derived transverse measurements as reference standards, we developed, validated, and compared multiple deep learning (DL) models to assess the feasibility of identifying MTD from frontal intraoral photographs.

**Methods:**

This study included 826 internal and 192 external patients who underwent paired frontal intraoral photographs and CBCT. MTD was determined based on the University of Pennsylvania analysis (UPA) and Yonsei transverse analysis (YTA) labels. DenseNet 121, ResNet 18, EfficientNet B0/B3, and MobileNetV3 Small/Large were trained separately on photographs using UPA- and YTA-based labels. Five-fold cross-validation was employed, and performance was evaluated on the internal and external test sets using accuracy, precision, recall, and F1 scores, along with confusion matrices and areas under the receiver operating characteristic curves. DeLong’s test assessed the differences between the models.

**Results:**

In the external test set under the UPA labelling scheme, ResNet 18 achieved the highest accuracy (90.62%). Under the YTA labelling scheme, DenseNet 121 and ResNet 18 achieved the highest accuracy (96.88%). Across all internal and external test sets using both labelling schemes, DenseNet 121 and ResNet 18 yielded the best overall performance, and no statistically significant difference was observed between the two models (*P* > .05).

**Conclusions:**

The DL models demonstrated strong potential for analysing frontal intraoral photographs to detect MTD. These findings provide initial insights into the use of DL models to identify MTD from frontal intraoral photographs for orthodontic purposes.

**Clinical relevance:**

This study demonstrates the feasibility of using DL-based recognition of frontal intraoral photographs to identify MTD. As a cost-effective adjunctive tool, the proposed approach may assist clinicians in identifying MTD and help improve case selection for CBCT imaging.

## Introduction

In recent years, artificial intelligence (AI) has advanced rapidly in dentistry and is increasingly recognised as a valuable tool for improving clinical efficiency and the consistency of decision-making.^1^ Orthodontic diagnosis and treatment planning rely on multimodal data – including photographs, cone-beam computed tomography (CBCT), lateral cephalometric analyses, and intraoral scans – thereby increasing the need for data integration and quantitative assessment throughout diagnosis, treatment planning, and outcome evaluations.^2^ As datasets continue to expand and computing power increases, machine learning models have been progressively incorporated into orthodontic decision-support workflows, with deep learning (DL) models, based on multilayer neural networks, showing particular promise.^3^ By learning representations directly from raw data, DL has demonstrated strong potential across image analysis and diagnosis,^4^ automated treatment planning,^5^ facial recognition and analysis,^6^ predictive modelling,^7^ and patient monitoring,^8^ thereby providing a robust technical foundation for AI applications in orthodontics.

Maxillary transverse deficiency (MTD) is frequently observed in orthodontic practice, with a reported prevalence of 8% to 23%.^9^ From a dentofacial and occlusal perspective, MTD most commonly manifests as unilateral or bilateral posterior crossbite, dental crowding, and a constricted V-shaped maxillary arch, and may also be accompanied by functional mandibular shifts and facial asymmetry.^10^^,^^11^ Beyond these craniofacial and dental findings, MTD has also been associated with narrow nasal passages, which may increase nasal airway resistance and predispose patients to mouth breathing or obstructive sleep apnoea.^12^ Consequently, the precise identification of MTD remains a critical prerequisite for formulating an effective orthodontic strategy and ensuring its comprehensive management.

A wide range of approaches and measurements have been proposed for diagnosing MTD.^13^^,^^14^ As CBCT has become increasingly integrated into orthodontic diagnosis and treatment planning, it is now used more frequently to assess MTD.^15^ CBCT-based transverse evaluation methods include the University of Pennsylvania analysis (UPA),^16^ the Case Western Reserve University analysis,^17^ the Yonsei transverse analysis (YTA),^18^ and the Miner analysis.^19^ Although there is no universally accepted ‘gold standard’ for diagnosing MTD,^20^ UPA and YTA have attracted increasing attention in recent years in both clinical practice and research.^11^ Feştilă et al^21^ assessed the diagnostic accuracy of Pont’s Index for identifying MTD by comparing its findings with the UPA, which served as the reference standard. Zhang et al^22^ evaluated the reliability of the YTA for identifying MTD and found that, compared with Andrews’ Element III analysis, YTA demonstrated higher intra and interexaminer reliability and greater diagnostic agreement. Collectively, these findings support the growing role of UPA and YTA as clinically relevant reference frameworks for MTD assessment.^10^

Although UPA and YTA have gained increasing attention, their implementation remains inherently dependent on CBCT-derived measurements for the diagnosis of MTD. For patients, obtaining CBCT images may increase the radiation exposure and incur additional financial costs. Interpreting CBCT datasets, identifying anatomical landmarks, and performing subsequent measurements can be time-consuming and technically demanding for clinicians. In contrast, standardised frontal intraoral digital photographs are routinely collected as part of orthodontic records and are readily available at a relatively low cost. The classic approach to visual evaluation is increasingly being augmented by AI-driven technologies.^23^ Accordingly, using CBCT-derived transverse measurements as the reference standard, DL models can be developed and validated to identify MTD from frontal intraoral photographs, thereby providing a photograph-based screening or adjunctive tool and reducing reliance on CBCT.

To date, to our knowledge, the performance of DL models for identifying MTD from frontal intraoral photographs using CBCT as the reference standard has not been established. Therefore, in this study, we aimed to preclassify samples according to CBCT-derived transverse measurements, validate DL models for photograph-based classification, and compare the diagnostic performance of different DL architectures while evaluating their potential clinical utility.

## Material and methods

### Internal dataset

**Fig. 1:**
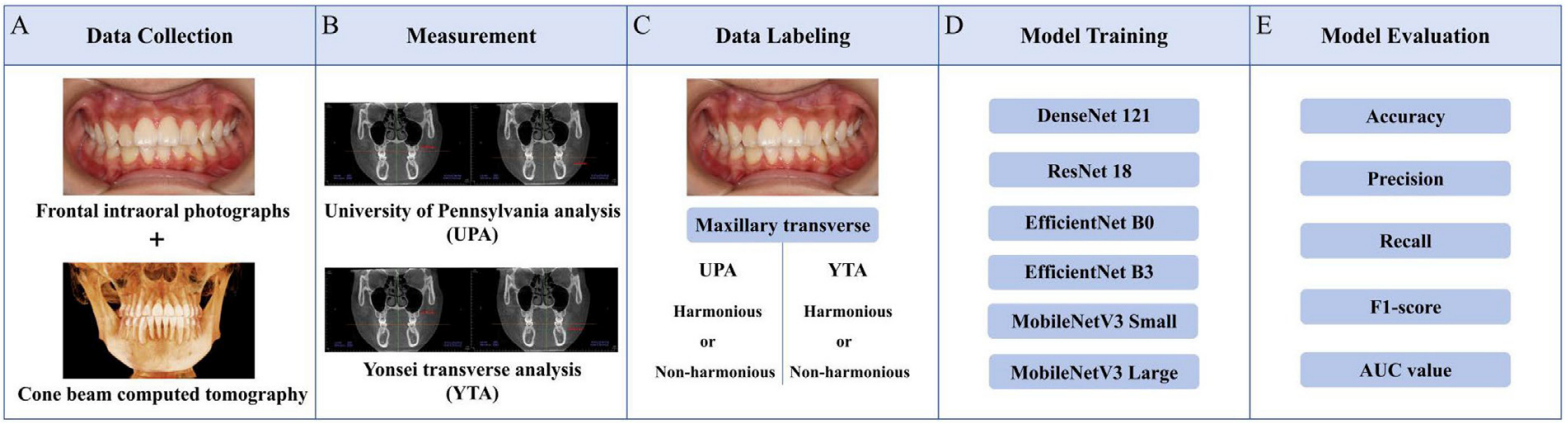
Overview of the study design and workflow.

This study was conducted in accordance with the Checklist for Artificial Intelligence in Medical Imaging recommendations,^24^ and the complete research workflow is illustrated in Figure 1. This retrospective study was reviewed and approved by the Medical Ethics Committee of the Stomatological Hospital, Tianjin Medical University (No. TMUhMEC20260216). The dataset was retrieved from the clinical database of the Department of Orthodontics at Tianjin Medical University Stomatological Hospital (Tianjin, China). The inclusion period spanned from January 2022 to October 2025 and covered all initial orthodontic consultations within this timeframe. Participants were eligible for inclusion if they were aged ≥16 years with complete permanent dentition, specifically requiring fully erupted maxillary and mandibular first permanent molars. A stringent set of exclusion criteria were applied to ensure the integrity of the study sample. Patients were excluded if they presented with (1) extensive tooth defects such as buccal caries or buccal wedge-shaped defects in any of the first permanent molars, (2) radiographic signs of periodontal disease, (3) missing first permanent molars, (4) a history of systemic conditions or pathologies, or (5) a history of prior or current orthodontic treatment.

The final dataset consisted of paired records from 826 patients, comprising frontal intraoral photographs and their corresponding CBCT scans; these imaging data were obtained from patients who required three-dimensional diagnostic assessments for various clinical indications. To maintain high standards of bioethics and patient confidentiality, all datasets were completely deidentified prior to the computational analysis and model training phases.

Frontal intraoral photographs simultaneously capture the maxillary and mandibular dentitions and the surrounding soft tissues in a single image, providing a more intuitive representation of the interarch occlusal relationship and allowing easier image acquisition. Therefore, frontal intraoral photographs were selected as the input modality in this study.

All photographs were captured in a dedicated photography suite within the Department of Orthodontics by three experienced orthodontic nurses, each with over 9 years of clinical expertise. A standardised imaging protocol was strictly implemented: patients were seated in a fixed dental chair with the head maintained in a stable position to ensure a consistent orientation and magnification across all sessions. A high-resolution digital single-lens reflex camera (Canon EOS 70D; Canon Inc.), equipped with a 100-mm macro lens and a ring flash, was utilised to ensure uniform illumination and a consistent depth of field. The aperture was set to f/25 with a shutter speed of 1/80 s and an ISO of 100, and an auxiliary fill light was used as needed. Frontal intraoral photographs were obtained while the patients were at maximum intercuspation, after dental surfaces were cleaned and air-dried to eliminate moisture-induced reflections. The resulting photographs had a resolution of approximately 4000 × 3000 pixels, preserving intricate morphological details.

All CBCT scans were performed using a KaVo 3D eXam CBCT scanner (KaVo Dental). To maintain high image fidelity and to minimise artefacts, all participants were required to remove metal objects before scanning. The imaging protocol was standardised using the following parameters: 120 kV, 5 mA; exposure time, 14.7 s, slice thickness, 0.25 mm. During the procedure, patients were seated with the Frankfort horizontal plane oriented parallel to the floor. They were instructed to maintain maximum intercuspation and remain completely stationary to ensure spatial accuracy of the scans. CBCT data in the Digital Imaging and Communications in Medicine format were imported into Invivo 6 (Anatomage Inc.) for multiplanar reformation and standardised head orientation.

### External dataset

To further evaluate the generalisability of the proposed models, an external validation dataset was introduced. Independent testing was performed using the publicly available FDTooth dataset,^25^ which includes frontal intraoral photographs with corresponding CBCT images. Given that the FDTooth dataset was originally collected for anterior fenestration and dehiscence detection, images were acquired to ensure clear visualisation of the anterior teeth and gingiva; consequently, in a subset of photographs, the molar-region teeth and surrounding soft tissues were slightly blurred or incompletely captured. Such cases were excluded. In addition, cases showing orthodontic brackets, resin attachments for clear aligner therapy, traction hooks, or temporary anchorage devices were excluded. Cases with posterior restorations and CBCT artifacts that could compromise image interpretation were also excluded. The trained models were then evaluated on this external dataset without any further training.

### CBCT measurement

To ensure high diagnostic accuracy, all CBCT landmarks were independently digitised by two senior clinicians, each possessing a minimum of 10 years of specialised experience in orthodontics. To minimise bias, both observers were blinded to each other’s results and the experimental grouping. Interobserver consistency was quantified via the intraclass correlation coefficient (ICC) based on a two-way mixed-effects model (absolute agreement). The observed ICC values (0.947-0.973) indicated a high degree of reliability according to established criteria. Consequently, the arithmetic mean of the two sets of measurements was recorded as the final value for subsequent data processing.

### UPA measurement procedure

**Fig. 2:**
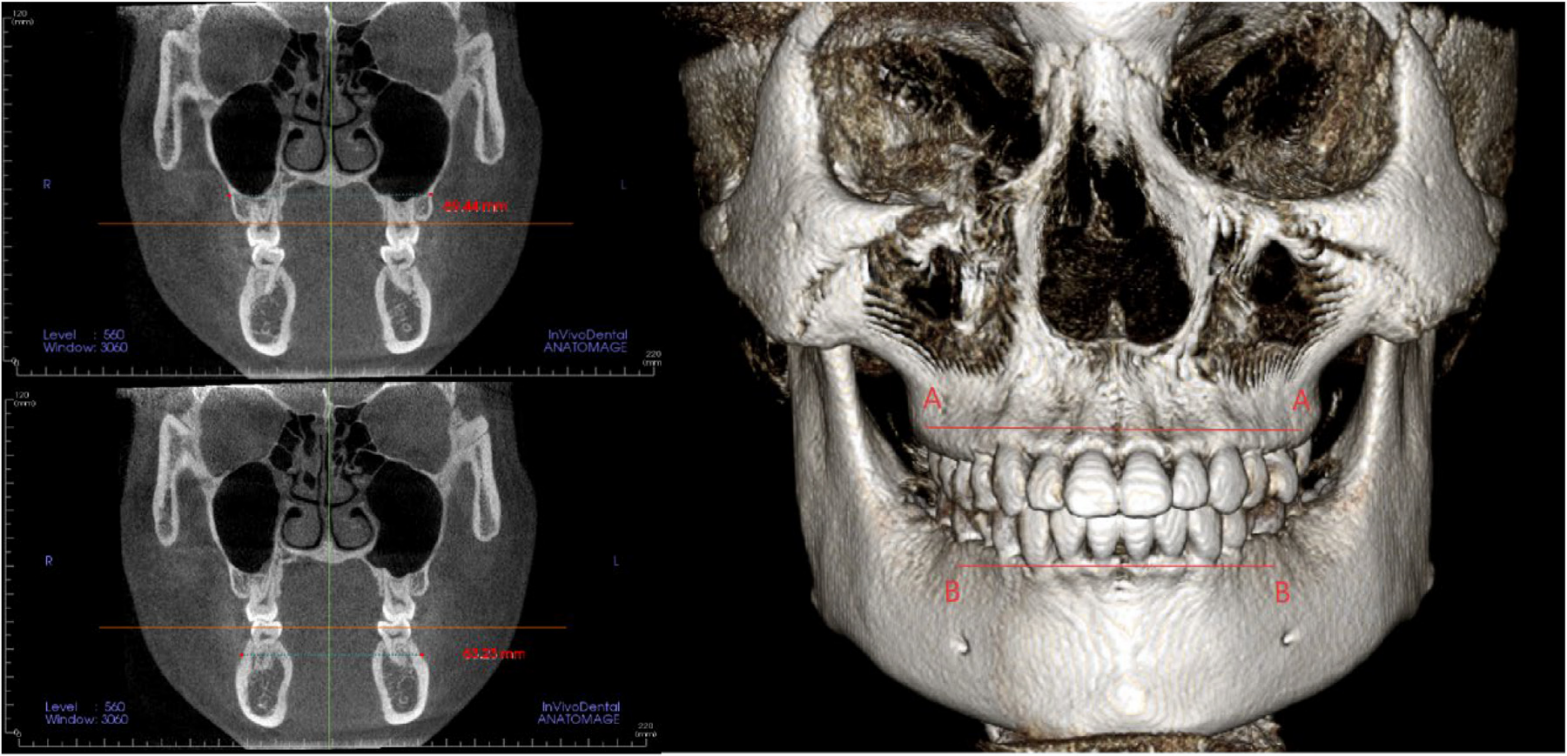
Schematic illustration of the University of Pennsylvania analysis measurement procedure.

After CBCT multiplanar reconstruction and standardised head reorientation, the maxillary and mandibular basal widths were quantified in the same measurement plane, and the bilateral landmarks were identified in this plane. Point A was placed at the junction of the maxillary tuberosity and zygomatic buttress, that is, at the intersection between the maxillary basal bone and the deepest concavity of the lateral maxillary contour. Point B was located by constructing a line passing through the resistance centres of the bilateral mandibular first molars and marking the intersection between this line and the outermost border of the buccal cortical bone. The maxillary basal width (A-A) and mandibular basal width (B-B) were obtained as the linear distances between the right and left A points and between the right and left B points, respectively. UPA was then calculated as UPA = (A-A) − (B-B). Values < 5 mm was interpreted as a maxillary transverse width deficiency. A schematic of the measurement setup is shown in Figure 2.

### YTA measurement procedure

**Fig. 3:**
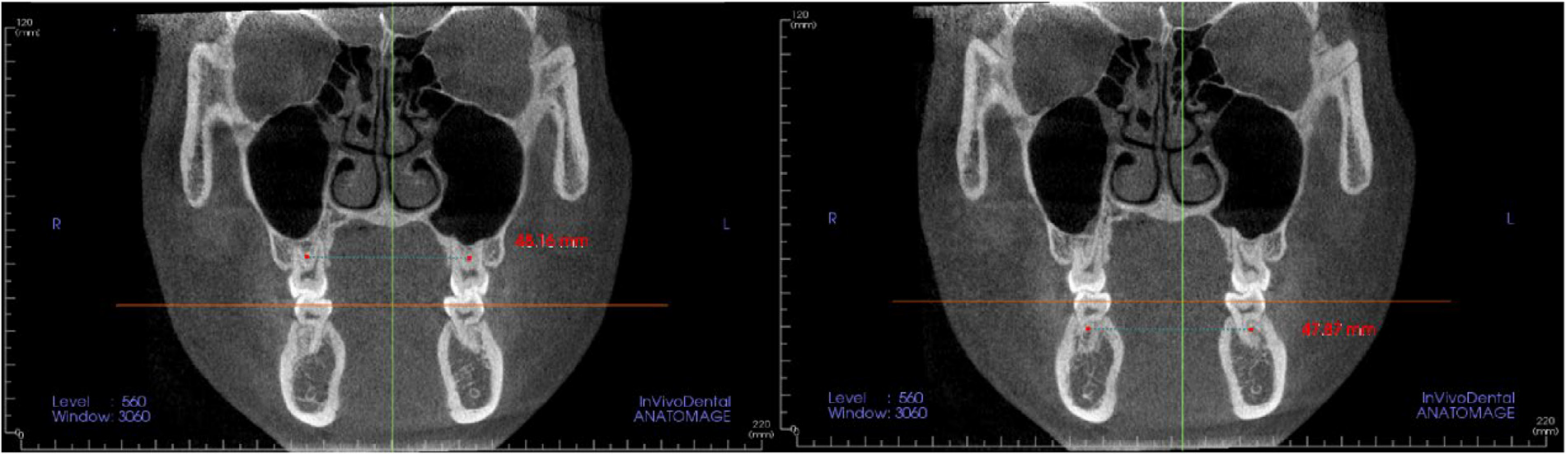
Schematic illustration of the Yonsei transverse analysis measurement procedure.

Following standardised head reorientation, the resistance centres of the first permanent molars were used as stable reference points for the transverse assessment. The resistance centres of the bilateral first molars were identified in both arches, and the maxillary and mandibular intermolar resistance centre widths were measured within the same reference plane system. The transverse maxillomandibular relationship was expressed as the interarch difference in the resistance-centre width (maxillary width minus mandibular width). Values < −2.26 mm were interpreted as MTD. A schematic of the measurement setup is shown in Figure 3.

### Image preprocessing

Before model training, all photographs underwent standardised image preprocessing. Images were manually reviewed for quality control, and photographs with severe blur, incomplete exposure of the posterior segments, or major artefacts were excluded. The remaining images were then manually cropped to centre the intraoral field, removing irrelevant peripheral regions while preserving the overall occlusal view and surrounding anatomical context. No manual landmark annotation was used during image preprocessing.

### Classification models

Several DL models that have been previously proven effective in image classification tasks were employed.^26^^,^^27^ Multiple representative backbones spanning different design families were evaluated to reduce the selection bias associated with any single architecture.

#### DenseNet 121

DenseNet 121, proposed by Huang et al,^28^ utilises a dense connectivity pattern in which each layer receives feature maps from all preceding layers as concatenated inputs within feed-forward dense blocks. This architecture facilitates extensive feature reuse and alleviates the vanishing gradient problem by strengthening feature propagation. By combining four sequential dense blocks with transition layers, the 121-layer model optimises parameter efficiency while enhancing representative power through continuous information integration across the network hierarchy.

#### ResNet 18

ResNet 18, introduced by He et al,^29^ employs a residual learning framework via shortcut connections to mitigate the degradation problem in deep networks. While deeper variants such as ResNet 50 or ResNet 101 offer higher representational capacity, ResNet 18 was selected in this study to achieve an optimal trade-off between accuracy and computational resource consumption. By minimising the total number of floating-point operations and the parameter volume, this 18-layer architecture ensures rapid inference and training stability while maintaining a robust feature extraction capability suitable for the target task.

#### EfficientNet B0 and EfficientNet B3

Proposed by Tan and Le,^30^ EfficientNet utilises a principled compound scaling method to uniformly balance network depth, width, and resolution. The architecture is built upon a Mobile Inverted Bottleneck Convolution block integrated with squeeze-and-excitation modules to enhance channel-wise feature dependencies. By evaluating the baseline B0 model alongside the scaled-up B3 variant, we aimed to leverage the optimised trade-offs between accuracy and total floating-point operations. Consequently, these models provide a robust framework that achieves superior feature representation while maintaining high efficiency across varying resource constraints.

#### MobileNetV3 small and MobileNetV3 large

Proposed by Howard et al,^31^ MobileNetV3 is a highly efficient architecture specifically designed for next-generation mobile inferences. The model utilises enhanced inverted residual blocks integrated with squeeze-and-excitation modules and an h-swish activation function to optimise the feature representation. Although the large variant targets high-performance requirements, the small version is streamlined for deployment in environments with minimal computational resources. These two configurations offer scalable and low-latency solutions for diverse real-time computer-vision tasks.

### Model training

All models were implemented in PyTorch 2.0.1 (PyTorch Foundation) for the binary classification of MTD using a unified training protocol. All experiments were conducted on the Google Colab Pro environment, with model training accelerated by an NVIDIA H100 GPU (approximately 80 GB VRAM). Specifically, each of the six DL models was trained on the internal frontal intraoral photograph datasets using the UPA and YTA labelling protocols, resulting in 12 distinct training sessions. Transfer learning was applied to all architectures by initialising them with ImageNet-pretrained weights and replacing the original classifier with a task-specific, fully connected layer with softmax outputs. Images were resized to 224 × 224 pixels^32^ and normalised using ImageNet standardisation parameters. To improve model generalisation while preserving dental structural features, mild data augmentation techniques were applied during training, including horizontal flipping, small-angle rotation within ±20°, and random brightness and contrast adjustments within ±20%. The models were optimised using the Adam optimiser with cross-entropy loss and weight decay. Architecture-specific learning rates and batch sizes were used with a validation-driven adaptive learning rate scheduler. Training was performed for up to 50 epochs with early stopping based on the validation F1 score, and the best-performing checkpoint was retained. The internal datasets were evaluated using stratified 5-fold cross-validation to ensure robust estimation of model performance.^33^ In each fold, approximately 80% of the data were used for training and the remaining 20% were used for validation. The training process was repeated across all five folds, and the best-performing model checkpoint from each fold was retained. To further assess the statistical stability of the evaluation metrics, bootstrap resampling was applied to estimate 95% confidence intervals (95% CI) for the main performance indicators.

### Model evaluation

MTD classification performance was evaluated on both the internal test dataset and an independent external test dataset to assess accuracy and generalisability across different data sources. Accuracy, precision, recall, and F1 score were used as the primary evaluation metrics. Confusion matrices were generated to visualise diagnostic performance for harmonious and nonharmonious maxillary transverse relationship samples. Receiver operating characteristic (ROC) curves were plotted to compare model performance across decision thresholds, and the areas under the ROC curves (AUCs) were calculated as a threshold-independent measure of discrimination. Sample-level behaviour was examined by visualising probability outputs from different models using the same test cases. Radar plots were used to summarise evaluation metrics and facilitate cross-model comparisons of performance. To statistically compare the discriminative performance between models, pairwise comparisons of the AUCs were conducted using DeLong’s test for correlated ROC curves.

## Results

### Dataset

In the internal dataset (*n* = 826), the included patients ranged in age from 16 to 39 years; 323 were male (39.10%) and 503 were female (60.90%). In the external dataset (*n* = 192), the age range was 9 to 55 years, with 64 males (33.33%) and 128 females (66.67%).

For the internal dataset, among the 826 included frontal intraoral photographs, UPA-based labelling classified 407 images (49.27%) as demonstrating a harmonious maxillomandibular transverse width relationship and 419 images (50.73%) as nonharmonious. Using YTA, 480 images (58.10%) were categorised as harmonious, whereas 346 images (41.90%) were categorised as nonharmonious.

For the external dataset, among the 192 included frontal intraoral photographs, UPA-based labelling classified 148 images (77.08%) as harmonious and 44 images (22.92%) as nonharmonious. Using YTA, 132 images (68.75%) were categorised as harmonious and 60 images (31.25%) as nonharmonious. The demographic and clinical characteristics of the study cohort are summarised in Table 1.

**Table 1:** Demographic and clinical characteristics of included patients.

Characteristic	Internal dataset (*n* = 826)		External dataset (*n* = 192)
	Training (*n* = 660)	Test (*n* = 166)	
Age, y	21.29 ± 3.39	21.32 ± 2.86	25.34 ± 7.15
Sex			
Male	256 (38.79%)	67 (40.36%)	64 (33.33%)
Female	404 (61.21%)	99 (59.64%)	128 (66.67%)
Distribution (UPA)			
Harmonious	325 (49.24%)	82 (49.40%)	148 (77.08%)
Nonharmonious	335 (50.76%)	84 (50.60%)	44 (22.92%)
Range (UPA), mm			
Harmonious	5.03 to 9.46	5.02 to 8.89	5.08 to 8.38
Nonharmonious	−5.49 to 4.98	−5.14 to 4.97	−3.14 to 4.89
Measurement (UPA), mm			
Harmonious	6.01 ± 1.10	6.19 ± 0.98	5.98 ± 1.02
Nonharmonious	1.26 ± 2.79	1.33 ± 3.07	1.05 ± 2.35
Distribution (YTA)			
Harmonious	384 (58.18%)	96 (57.83%)	132 (68.75%)
Nonharmonious	276 (41.82%)	70 (42.17%)	60 (31.25%)
Range (YTA), mm			
Harmonious	−2.21 to 6.65	−2.24 to 6.08	−2.07 to 3.88
Nonharmonious	−4.49 to −2.28	−4.35 to −2.34	−4.08 to −2.31
Measurement (YTA), mm			
Harmonious	1.03 ± 2.08	1.30 ± 2.45	0.97 ± 1.29
Nonharmonious	−3.34 ± 0.77	−3.25 ± 0.70	−3.04 ± 0.60

Values are *n* (%), mean±standard deviation, or range (minimum–maximum).

UPA, the University of Pennsylvania analysis; YTA, the Yonsei transverse analysis.

### UPA-based labelling

Under UPA-based labelling in the internal test dataset, DenseNet 121 outperformed the other models, achieving a mean accuracy of 93.82%. In contrast, EfficientNet B3 yielded the lowest mean accuracy (81.96%). In terms of discriminative performance, DenseNet 121 attained the highest AUC (97.63%), whereas EfficientNet B0, EfficientNet B3, and MobileNetV3 Large showed comparatively lower AUCs, all below the 90% threshold. In the external test dataset, ResNet 18 achieved the highest mean accuracy (90.62%). DenseNet 121 showed a comparable mean accuracy (89.58%), closely approaching that of ResNet 18. Consistently, the two models exhibited similar discriminative ability, with AUCs of 95.33% for ResNet 18 and 94.96% for DenseNet 121 (Table 2).

**Table 2:** Model accuracy when using University of Pennsylvania analysis labels.

	Accuracy (%)	Precision (%)	Recall (%)	F1-score (%)	AUC value (%)
Internal test dataset					
DenseNet 121	93.82 ± 1.46	92.30 ± 3.05	95.95 ± 1.99	94.05 ± 1.34	97.63 (96.64-98.44)
ResNet 18	92.98 ± 0.92	89.71 ± 1.90	97.38 ± 1.30	93.37 ± 0.83	97.08 (95.95-98.06)
EfficientNet B0	84.50 ± 2.59	86.95 ± 3.82	81.86 ± 2.26	84.30 ± 2.41	89.69 (87.57-91.77)
EfficientNet B3	81.96 ± 1.02	82.81 ± 3.80	81.85 ± 6.32	82.09 ± 1.61	87.48 (85.01-89.83)
MobileNetV3 Small	90.07 ± 1.75	89.97 ± 3.29	90.69 ± 3.33	90.25 ± 1.72	96.00 (94.77-97.09)
MobileNetV3 Large	82.93 ± 2.37	82.28 ± 4.28	84.95 ± 4.08	83.47 ± 2.17	89.92 (87.68-92.07)
External test dataset					
DenseNet 121	89.58	68.75	100.00	81.48	94.96 (91.60-97.59)
ResNet 18	90.62	80.95	77.27	79.07	95.33 (92.18-97.90)
EfficientNet B0	75.00	45.45	45.45	45.45	62.04 (52.18-71.94)
EfficientNet B3	65.62	34.29	54.55	42.11	64.74 (55.60-74.00)
MobileNetV3 Small	75.00	47.83	100.00	64.71	92.66 (88.91-95.86)
MobileNetV3 Large	82.29	60.87	63.64	62.22	87.78 (82.09-92.67)

Values are mean±standard deviation, or *n* (95%CI).

AUC, area under the receiver operating characteristic curve; 95%CI, 95% confidence intervals.

**Fig. 4:**
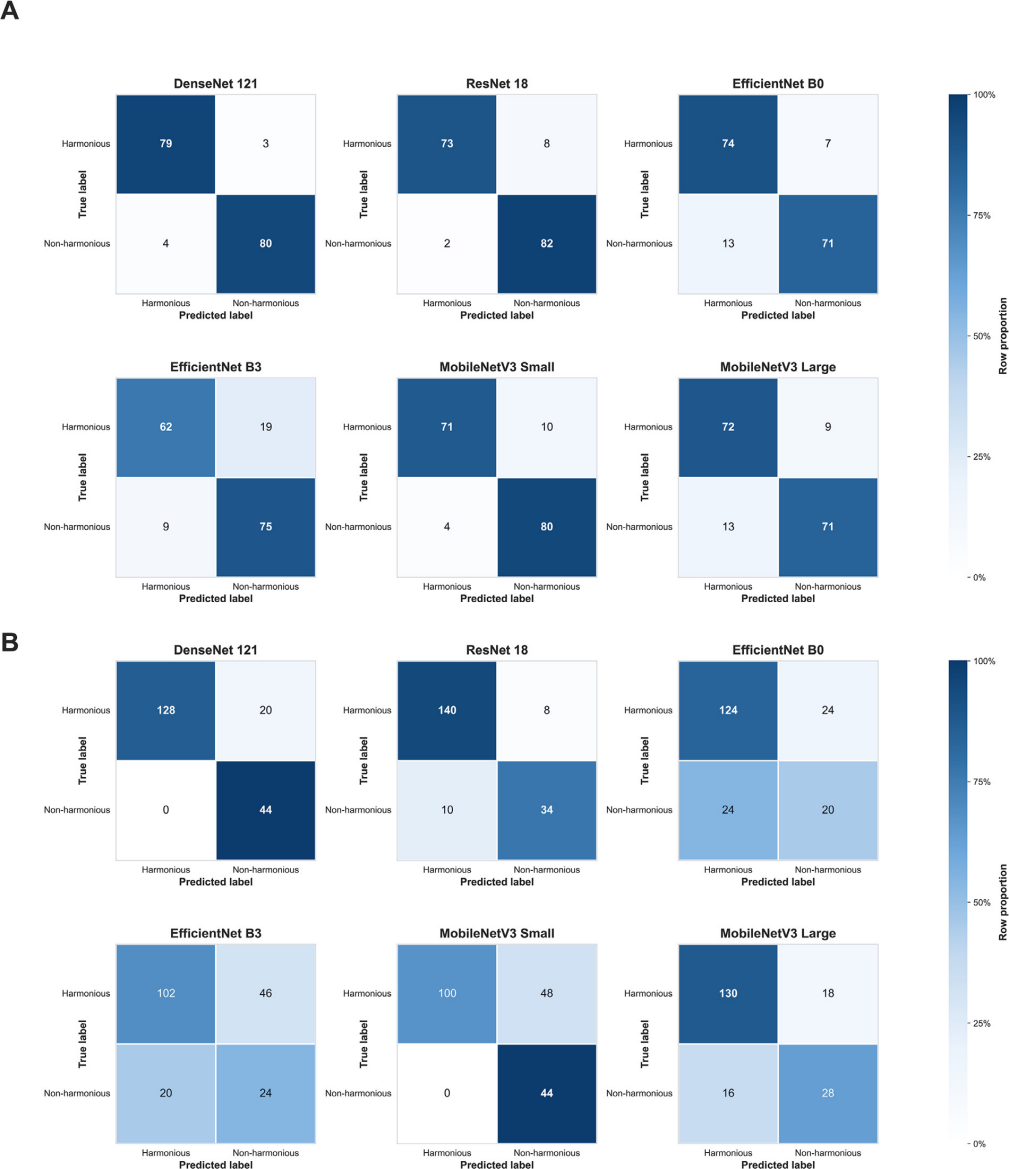
Confusion matrices of the evaluated models using the University of Pennsylvania analysis (UPA) labelling method: (A) internal test set; (B) external test set.

The confusion matrices under the UPA-based labelling scheme exhibit generally consistent classification patterns across models. In the internal dataset, most predictions were concentrated along the main diagonal, indicating effective discrimination between harmonious and nonharmonious samples, with relatively fewer misclassifications observed for DenseNet 121 and ResNet 18 (Figure 4A). In the external dataset, a similar overall trend was observed, although the distribution of misclassifications varied among models (Figure 4B). The ROC curves and corresponding AUC values further illustrate the discriminative performance of the models (Supplementary 1). Overall, most models achieved strong discriminative performance in the internal dataset, while slightly larger performance differences were observed in the external validation dataset.

### YTA-based labelling

Under YTA-based labelling in the internal test dataset, DenseNet 121 and ResNet 18 emerged as the top-performing models, each achieving a mean accuracy of 99.15%. EfficientNet-B0 was the only model with a mean accuracy below 90%, achieving a mean accuracy of 84.02%. Although DenseNet 121, ResNet 18, and MobileNetV3 Small all achieved superior discriminative performance, with AUC values exceeding 99%, their effectiveness across other evaluation metrics was not uniform. In particular, MobileNetV3 Small underperformed compared to the other two models in terms of Accuracy, Precision, Recall, and F1-score.

In the external test dataset, both DenseNet 121 and ResNet 18 achieved an average accuracy of 96.88%, outperforming all other models. DenseNet 121 recorded an AUC of 97.78%, slightly surpassing the 97.27% achieved by ResNet 18. While MobileNetV3 Small also reached an AUC of 97.78%, it failed to match the performance of DenseNet 121 and ResNet 18 across the remaining four metrics. Notably, despite its superior accuracy, DenseNet 121 yielded average Precision and F1-score values below the 90% threshold (Table 3).

**Table 3:** Model accuracy when using Yonsei transverse analysis labels.

	Accuracy (%)	Precision (%)	Recall (%)	F1-score (%)	AUC value (%)
Internal test dataset					
DenseNet 121	99.15 ± 0.54	98.02 ± 1.26	100.00 ± 0.00	99.00 ± 0.64	99.41 (98.79-99.90)
ResNet 18	99.15 ± 0.81	98.04 ± 1.87	100.00 ± 0.00	99.00 ± 0.95	99.69 (99.41-99.92)
EfficientNet B0	95.03 ± 1.57	93.82 ± 3.67	94.51 ± 1.90	94.12 ± 1.81	98.31 (97.39-99.01)
EfficientNet B3	84.02 ± 7.19	81.21 ± 10.33	81.53 ± 7.05	81.20 ± 8.02	89.92 (87.82-91.91)
MobileNetV3 Small	98.06 ± 0.66	95.84 ± 0.97	99.71 ± 0.65	97.73 ± 0.78	99.08 (98.39-99.66)
MobileNetV3 Large	92.74 ± 4.08	90.70 ± 4.21	92.24 ± 8.04	91.32 ± 5.18	97.29 (96.33-98.21)
External test dataset					
DenseNet 121	96.88	90.91	100.00	95.24	97.78 (95.17-99.61)
ResNet 18	96.88	90.91	100.00	95.24	97.27 (94.33-99.39)
EfficientNet B0	72.92	54.76	76.67	63.89	78.23 (70.48-85.38)
EfficientNet B3	63.54	43.90	60.00	50.70	63.89 (54.89-71.85)
MobileNetV3 Small	92.71	82.86	96.67	89.23	97.78 (95.28-99.57)
MobileNetV3 Large	86.46	74.29	86.67	80.00	92.75 (88.97-95.89)

Values are mean±standard deviation, or *n* (95%CI).

AUC, area under the receiver operating characteristic curve; 95%CI, 95% confidence intervals.

**Fig. 5:**
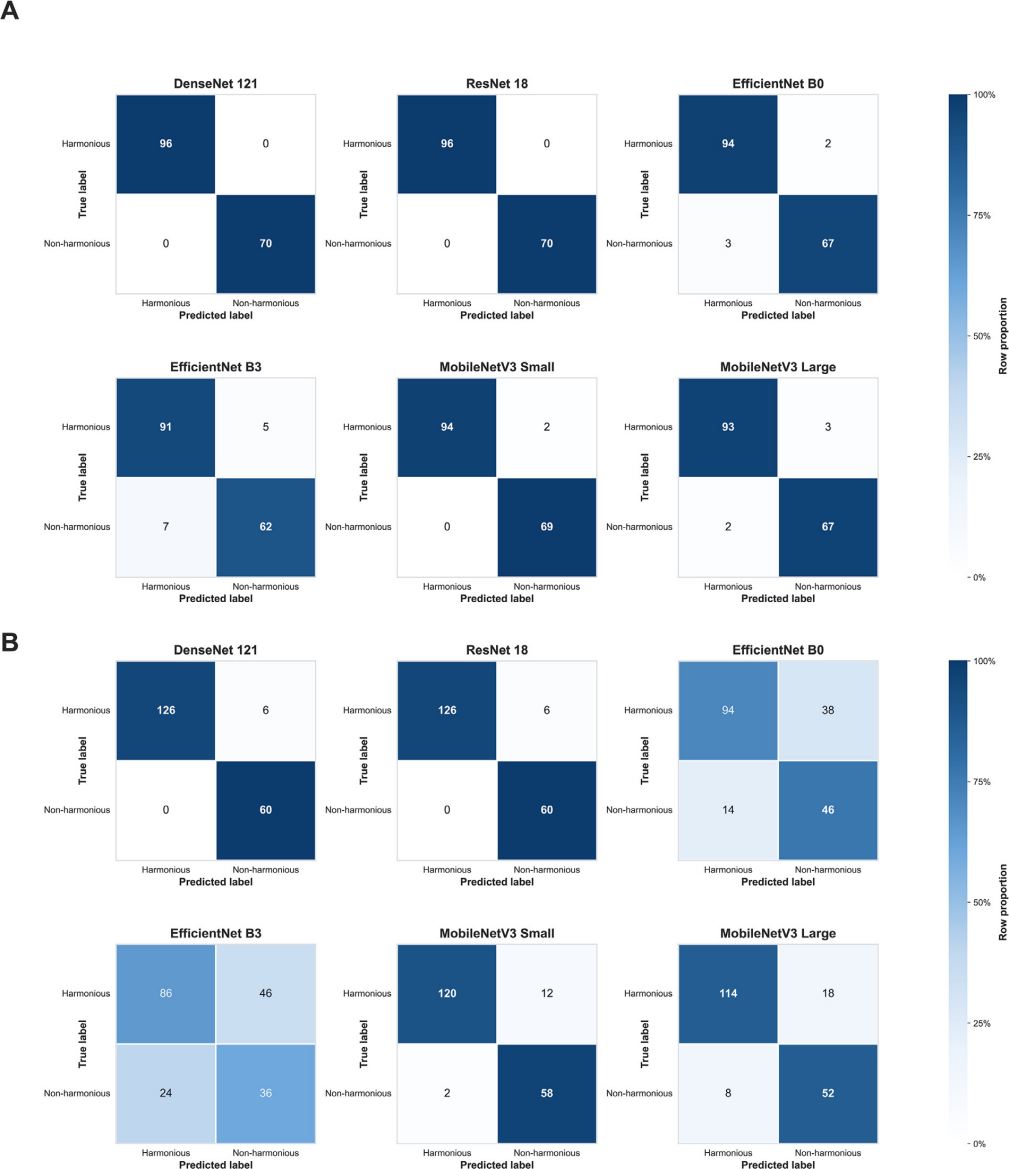
Confusion matrices of the models evaluated using the Yonsei transverse analysis (YTA) labelling method: (A) internal test set; (B) external test set.

The confusion matrices under the YTA-based labelling scheme demonstrate varying levels of classification consistency across models. Figure 5A shows the results in the internal dataset. Predictions from DenseNet 121 and ResNet 18 were mainly concentrated along the main diagonal, indicating stable classification performance, while the MobileNetV3 variants also showed relatively consistent results. In contrast, EfficientNet B0 and B3 exhibited greater class confusion in several cases. Figure 5B shows the results in the external dataset. A similar overall pattern was observed, although differences in misclassification among models became more apparent. The ROC curves and corresponding AUC values further illustrate the discriminative performance of the models (Supplementary 1). In general, most models showed strong discrimination on the internal dataset, whereas performance gaps among models were slightly more pronounced on the external dataset.

### Comparative analysis of UPA and YTA labelling

**Fig. 6:**
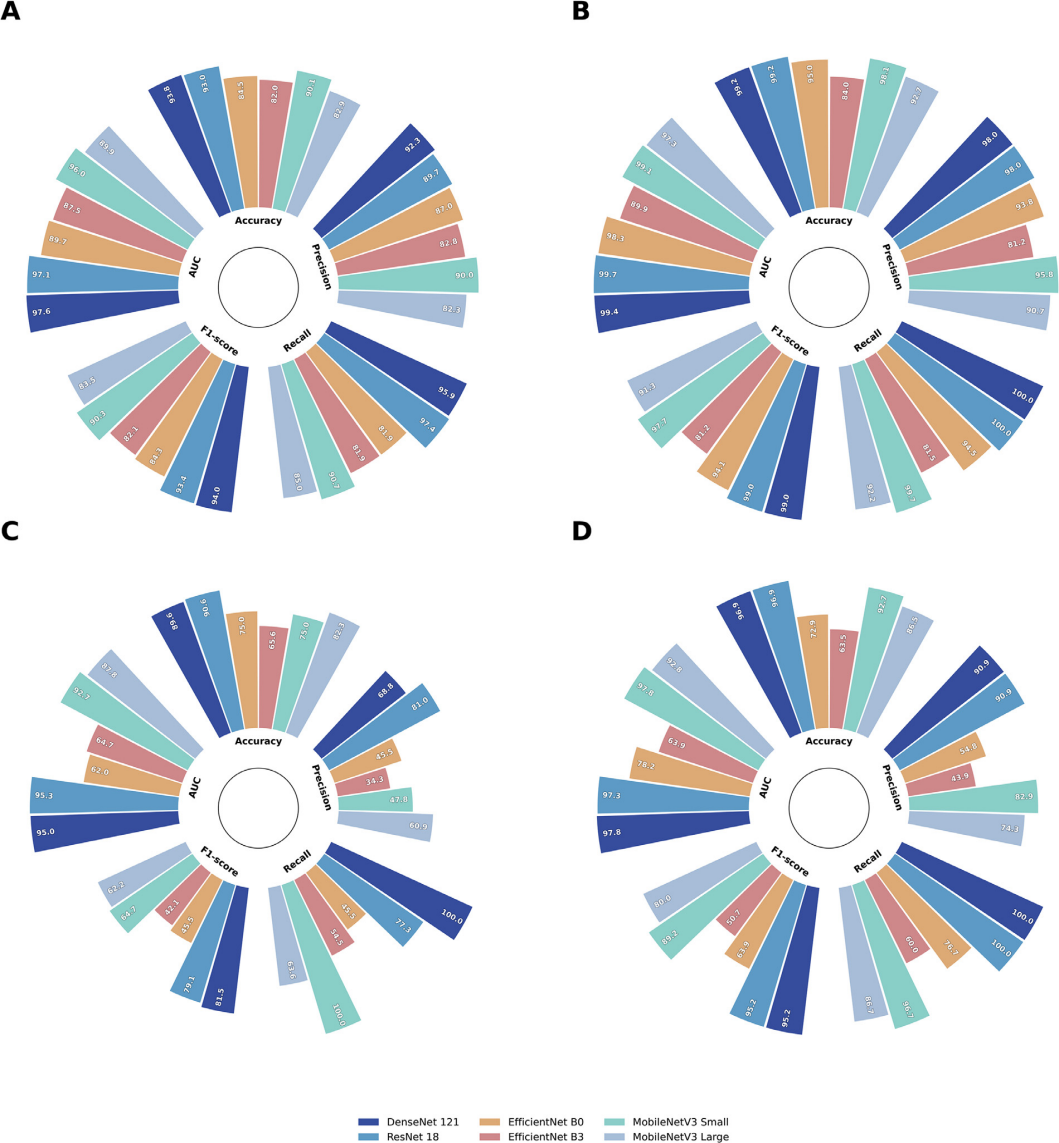
Multidimensional performance of University of Pennsylvania analysis (UPA) and Yonsei transverse analysis (YTA) across models on internal and external test sets: (A) internal–UPA; (B) internal–YTA; (C) external–UPA; (D) external–YTA.

Figure 6 summarises the performance of six models using radar plots of five evaluation metrics under the UPA and YTA labelling schemes. For the internal test set (Fig. 6, Fig. 6), the radar polygons were comparatively regular and near-circular, indicating a more consistent and balanced metric profile. By contrast, the external test set results (Fig. 6, Fig. 6) showed less regular shapes and a reduced overall extent, together with greater dispersion across metrics. These patterns suggested that model behaviour was more variable when evaluated on external data and that there remains scope to optimise performance under external conditions.

### DeLong’s test for model comparison

Pairwise comparisons of ROC curves were performed using DeLong’s test to evaluate statistical differences in AUC values between models, as shown in Supplementary 2. In the internal datasets, the reference models demonstrated significantly higher AUC values than several alternative architectures. Specifically, in the UPA-labelled internal dataset, DenseNet 121 significantly outperformed EfficientNet B0, EfficientNet B3, MobileNetV3 Large, and MobileNetV3 Small (all *P* < .01), while no significant difference was observed between DenseNet 121 and ResNet 18 (*P* = .176). In the YTA-labelled internal dataset, ResNet 18 showed comparable performance to DenseNet 121 (*P* = .122), but significantly outperformed EfficientNet B0, EfficientNet B3, MobileNetV3 Large, and MobileNetV3 Small (all *P* < .05). Similar trends were observed in the external test datasets.

## Discussion

MTD is a prevalent condition that is frequently encountered in orthodontic practice. In this study, we evaluated six DL architectures – DenseNet 121, ResNet 18, EfficientNet B0, EfficientNet B3, MobileNetV3 Small, and MobileNetV3 Large – and systematically compared their performance in identifying MTD from frontal intraoral photographs against performance when using CBCT-derived UPA and YTA measurements as reference standards. By providing an efficient diagnostic tool, this approach is expected to facilitate the rapid preliminary identification of MTD, thereby supporting clinicians in early decision-making.

Intraoral photographs acquired during orthodontic treatment constitute an important component of orthodontic records and serve as key sources of information for diagnosis.^34^ However, the vast majority of current research on DL in dentistry remains tied to radiological imaging. A 2025 review on the use of AI in dentistry further underscored this trend, reporting that imaging modalities such as CBCT and orthopantomograms were utilised in 84.4% of cases.^35^ Given that intraoral photographs represent a highly accessible and nonionising imaging modality, it is imperative to fully harness the potential of DL models to extract diagnostic insights from photographic records, thereby expanding their clinical utility.^36^

DL models can discern predictable mappings between photographic features and radiographic outcomes, a cross-modal relationship that has recently been reported in orthodontic diagnostics. For instance, Kartbak et al^37^ achieved accuracies as high as 99% in predicting internal cephalometric parameters, including the incisor mandibular plane angle, interincisal angle, U1–palatal plane, and Wits appraisal, solely from intraoral photographs. Although the aforementioned studies primarily relied on lateral cephalograms, the present work was based on CBCT; nevertheless, both approaches used quantitative image-derived measurements as training labels to develop DL models for cross-modal inference.

In this study, ResNet 18 achieved the highest accuracy of 90.62% with UPA-based labels in the external test set. With YTA-based labels, DenseNet 121 and ResNet 18 delivered the best performances, each achieving an accuracy of 96.88%. A growing body of research has leveraged intraoral photographs to develop and validate DL models for screening and subtype classification of common malocclusion patterns, thereby exploring their potential utility in clinical diagnosis. Chai et al^38^ developed and evaluated a DL model for the automated detection and classification of anterior crossbites using 1865 intraoral photographs, achieving a test-set accuracy of 0.965 and suggesting its potential utility for intraoral photograph-based anterior crossbite screening. Consistent with these studies, the present findings indicate that DL models using intraoral photographs can achieve strong performance in orthodontic diagnostic tasks, with the best-case accuracy herein exceeding 90%. In addition, Zhang et al^39^ developed a Swin Transformer – based model to automate the classification of occlusal types from intraoral photographs, achieving weighted F1 scores of approximately 0.89 to 0.90 across molars, canines, and incisors, indicating its potential to streamline occlusal assessment while reducing clinician subjectivity. Overall, these studies provide evidence that DL approaches using intraoral photographs have promising generalisability and clinical translational potential across screening, subtype classification, and occlusal relationship assessment for common malocclusions and may serve as a valuable adjunct to conventional orthodontic diagnostic workflows.

**Fig. 7:**
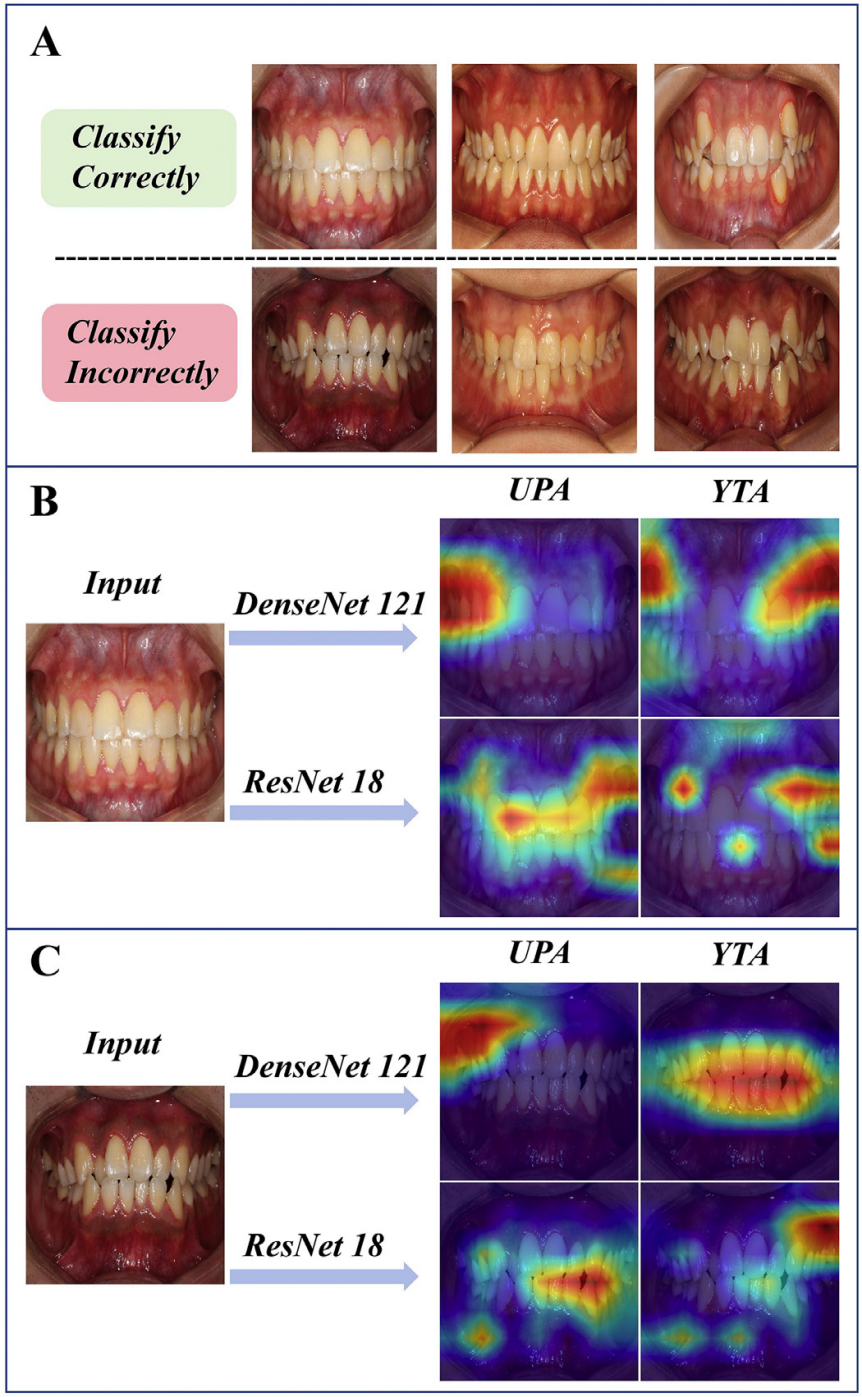
Schematic illustration of model classification results: (A) representative intraoral frontal photographs with correct and incorrect classifications; (B) correctly classified intraoral photographs and the corresponding heatmaps; (C) incorrectly classified intraoral photographs and the corresponding heatmaps.

Representative intraoral photographs from the test set illustrate both correct and incorrect classifications produced by the two top-performing models, DenseNet 121 and ResNet 18 (Figure 7A). In the correctly classified cases, the models achieved accurate predictions across diverse clinical presentations, including relatively well-aligned dentition, bilateral posterior crossbite, and the presence of ectopic teeth located outside the dental arch. In the misclassified cases, errors were mainly observed in milder or more localised conditions, such as slight crowding, isolated anterior crossbite involving individual incisors, and unilateral dental crowding. To enhance the interpretability of an MTD diagnosis derived solely from clinical photographs, we employed Gradient-weighted Class Activation Mapping^40^ to visualise the spatial attention of the DL models. This methodology facilitates the development of a transparent DL framework by superimposing colour-coded heat maps onto the input images, thereby identifying the specific spatial regions that most significantly influence the model’s diagnostic decisions.^40^ Figure 7B presents representative intraoral photographs that were correctly classified by both DenseNet 121 and ResNet 18 under the UPA- and YTA-based reference definitions, together with their corresponding heat maps. The saliency patterns differed between architectures: DenseNet 121 focused predominantly on the maxillary posterior segment, whereas ResNet 18 distributed attention more broadly across both the maxillary and mandibular dentitions. Despite their different attention patterns, both models were able to make correct predictions. Figure 7C shows a representative case that was misclassified by both models under both the UPA and YTA criteria, along with the corresponding heat maps. Clinically, the patient exhibited mild crowding with a partial anterior crossbite, and the maxillary molars demonstrated relative bilateral buccal inclination. Despite being classified as ‘harmonious’ based on CBCT measurements using both UPA and YTA, both models predicted ‘nonharmonious.’ The heat maps showed that both models primarily focused on the anterior crossbite region and the maxillary posterior molar region, suggesting that certain dentoalveolar features may have misled the models into making an erroneous transverse diagnosis.

Error analysis revealed consistent patterns among misclassified cases. False negatives occurred predominantly in borderline cases with UPA values close to the 5 mm diagnostic cutoff, underscoring the inherent ambiguity of transitional presentations. False positives were more common in cases with localised crowding or tooth rotations, in which a visually constricted arch did not reflect reduced skeletal width. In addition, buccolingual inclination of the maxillary molars can alter the apparent arch form and thereby obscure an underlying MTD. Dentoalveolar compensation may conceal a skeletal transverse deficiency, and posterior crossbite may be primarily dentoalveolar in origin. Although Noeldeke et al^41^ demonstrated that deep learning can detect visible posterior crossbite from intraoral images, crossbite visibility is not a reliable surrogate for skeletal MTD. Consistent with this, our heatmap analysis showed that the model’s MTD predictions were not driven solely by posterior features, suggesting that it integrates broader dentoalveolar morphological cues relevant to transverse relationships. Nevertheless, skeletal transverse deficiency masked by dentoalveolar compensation has not been systematically evaluated as an independent diagnostic dimension in this study. Future studies should specifically assess model performance in these subtle presentations to better define the diagnostic limits of intraoral photographs. As previously emphasised, clinical deployment of dental AI should remain cautious and evidence based, with AI positioned as decision support rather than a substitute for clinician judgement.^42^

The observed performance differences across models suggest that network architecture influences feature extraction and representation in MTD classification from frontal intraoral photographs. DenseNet 121 and ResNet 18 achieved accuracies above 89% with UPA-based labelling and above 96% with YTA-based labelling across both the internal and external datasets. Consistently, DeLong’s test indicated no statistically significant difference in AUC between the two models, supporting comparable discriminative performances. From an architectural perspective, DenseNet 121’s dense connectivity may promote feature reuse and improve gradient propagation, which could facilitate the capture of fine-grained patterns when visual cues in photographs are limited. ResNet 18’s strong performance may reflect the optimisation stability provided by residual connections, potentially contributing to robust generalisation across datasets. These observations are consistent with reports in other medical imaging tasks, including diabetic retinopathy detection and osteoporosis screening, where DenseNet 121 has outperformed architectures such as VGG16 and Inception V3, suggesting that it may better identify salient pathological signs in images.^43^ Additionally, ResNet 18 has been reported as an effective approach for predicting osteoporosis-related risk from lumbar spine radiographs.^44^ In contrast, EfficientNet B0 and MobileNetV3 showed performance declines, indicating difficulty in capturing critical features for accurate MTD classification. Although their compact designs facilitate deployment in resource-limited settings, such as on mobile devices,^45^ these efficiency-oriented architectures were insufficient for photo-based MTD identification in the present study.

Previous studies have suggested that UPA and YTA serve as more reliable and user-friendly screening tools in general practice.^46^ In this study, we observed that the diagnostic performance of DL models varied significantly when different diagnostic criteria were applied to operationalize MTD labelling. Specifically, across DenseNet 121, ResNet 18, MobileNetV3 Small, and MobileNetV3 Large, models trained with YTA-based labelling consistently achieved better performance than those trained with UPA labelling. One possible explanation is that YTA is based on the transverse discrepancy between maxillary and mandibular molar resistance-centre widths, which may correlate more directly with clinically visible intermolar and arch-form relationships captured in frontal intraoral photographs. By contrast, UPA incorporates basal skeletal measurements that may be less directly expressed in the visible dental appearance. Nonetheless, this interpretation requires further verification through multimodal studies and should not be overstated.

Collectively, the present findings suggest several potential, albeit preliminary, clinical implications. First, this approach may support preliminary screening for MTD using frontal intraoral photographs, helping clinicians identify patients who may warrant further evaluation for maxillary expansion and facilitating more individualised diagnostic workflows. Second, the proposed AI-based pipeline is best positioned as a decision-support adjunct rather than a definitive diagnostic tool, offering an additional layer of information to assist clinicians in triaging patients for comprehensive assessment when MTD is suspected. Importantly, CBCT selection should continue to be guided by clinical judgement and established indications. Third, mirroring the success of AI in photo-based occlusal classification,^47^ our approach shows promise for remote screening and virtual care. Initial assessment may be achieved using a single smartphone-acquired frontal intraoral occlusal photograph. This accessibility may facilitate earlier identification of MTD and timely intervention, potentially reducing subsequent treatment complexity and duration.

This study had certain limitations. First, the dataset was derived from patients who underwent CBCT for specific clinical indications rather than from an unselected screening population. This sampling frame may have inflated the apparent prevalence of MTD within the cohort, which in turn could lead to optimistically biased estimates of diagnostic performance. Therefore, caution is warranted when extrapolating these findings to general dental care settings where the true prevalence of MTD is likely lower. Future multicentre studies incorporating more diverse and representative cohorts into model training are needed to mitigate sampling bias and improve robustness across sites. Second, MTD assessment was based on UPA and YTA labels, which were used as training labels. Although both are clinically relevant and widely used transverse analyses, neither should be interpreted as an absolute gold standard. Future studies should incorporate additional model- or imaging-based transverse analysis methods for external validation to evaluate the robustness and consistency of models across different diagnostic frameworks. Third, diagnostic performance is highly sensitive to the quality and standardisation of the input images. Although high-resolution digital single-lens reflex cameras and standardised protocols were employed, clinical variations in camera angulation, soft-tissue retraction, and illumination remain potential sources of diagnostic variance.

To address these limitations, future efforts should prioritise the development of more robust algorithms, potentially through advanced data augmentation and domain adaptation techniques that can maintain high diagnostic accuracy despite variations in clinical photographs. In addition, subsequent studies will further evaluate the inclusion of occlusal and multiview intraoral photographs to determine whether they provide incremental performance gains beyond the current approach.

## Conclusions

To our knowledge, this study is among the first to attempt to directly identify MTD from frontal intraoral photographs using DL models. Our findings suggest that this AI-driven approach can provide clinicians with an efficient auxiliary tool for the preliminary and rapid screening of MTD, with significant potential to streamline clinical workflows. Nevertheless, it is intended to supplement clinical processes rather than replace conventional imaging techniques. Further clinical validation is required to fully realise its potential as a reliable adjunct to established diagnostic protocols.

## Author contributions

Conceptualisation: J.L. and Y.W. Methodology: J.L. and R.W. Data collection: J.L., Z.Y. and J.N. Data analysis: R.W. and J.L. Writing – original draft preparation: J.L. Writing – review and editing: Z.Y., Z.Z. and Y.W.

## Ethical approval and informed consent

The study was conducted in accordance with the Declaration of Helsinki and was reviewed and approved by the Medical Ethics Committee of the Stomatological Hospital, Tianjin Medical University (approval number: TMUhMEC20260216).

## Declaration of generative AI and AI-assisted technologies in the manuscript preparation process

Not applicable.

## Funding

This work was supported by the Tianjin Science and Technology Plan Project (No. 25YFXTHZ00490).

## Data availability

The data used in this study will not be shared as it contains sensitive patient information.

## Conflict of interest

None disclosed.

## Appendix Supplementary materials

Image, application 1

Image, application 2

## Footnotes

Supplementary material associated with this article can be found in the online version at doi:10.1016/j.identj.2026.109689.
