# Supplementary material for: Comparing Deep Learning Models for Identifying Maxillary Transverse Deficiency from Intraoral Photographs
Source: Int Dent J. 2026 Jun 19;76(4):109689. doi: 10.1016/j.identj.2026.109689 (PMC13314740; doi:10.1016/j.identj.2026.109689)
Supplement: Supplementary file 1 [file mmc1.docx]

**Supplementary 1**

Receiver Operating Characteristic (ROC) curves of the evaluated models.





A. ROC curves of the evaluated models were obtained in the internal dataset using the UPA labelling method.

B. ROC curves of the evaluated models were obtained in the external dataset using the UPA labelling method.

C. ROC curves of the evaluated models were obtained in the internal dataset using the YTA labelling method.

D. ROC curves of the evaluated models were obtained in the external dataset using the YTA labelling method.

ROC, Receiver Operating Characteristic

UPA, the University of Pennsylvania analysis

YTA, the Yonsei transverse analysis
