# Supplementary material for: Comparing Deep Learning Models for Identifying Maxillary Transverse Deficiency from Intraoral Photographs
Source: Int Dent J. 2026 Jun 19;76(4):109689. doi: 10.1016/j.identj.2026.109689 (PMC13314740; doi:10.1016/j.identj.2026.109689)
Supplement: Supplementary file 2 [file mmc2.docx]

**Supplementary 2**

Pairwise comparison of model AUC values using DeLong’s test.

| Evaluation set | Method | Reference model | Compared model | p-value |
| --- | --- | --- | --- | --- |
| Internal | UPA | DenseNet121 | EfficientNet-B0 | p < 0.001 |
|  |  |  | EfficientNet-B3 | p < 0.001 |
|  |  |  | MobileNetV3-Large | p < 0.001 |
|  |  |  | MobileNetV3-Small | p = 0.002 |
|  |  |  | ResNet18 | p = 0.176 |
|  | YTA | ResNet18 | DenseNet121 | p = 0.122 |
|  |  |  | EfficientNet-B0 | p < 0.001 |
|  |  |  | EfficientNet-B3 | p < 0.001 |
|  |  |  | MobileNetV3-Large | p < 0.001 |
|  |  |  | MobileNetV3-Small | p = 0.014 |
| External | UPA | ResNet18 | DenseNet121 | p = 0.755 |
|  |  |  | EfficientNet-B0 | p < 0.001 |
|  |  |  | EfficientNet-B3 | p < 0.001 |
|  |  |  | MobileNetV3-Large | p = 0.001 |
|  |  |  | MobileNetV3-Small | p = 0.120 |
|  | YTA | DenseNet121 | EfficientNet-B0 | p < 0.001 |
|  |  |  | EfficientNet-B3 | p < 0.001 |
|  |  |  | MobileNetV3-Large | p = 0.007 |
|  |  |  | MobileNetV3-Small | p = 1.000 |
|  |  |  | ResNet18 | p = 0.617 |

UPA, the University of Pennsylvania analysis;YTA, the Yonsei transverse analysis
